# Assessment of Labor Practices in Healthcare Using an Innovatory Framework for Sustainability

**DOI:** 10.3390/medicina59040796

**Published:** 2023-04-19

**Authors:** Flaviu Moldovan, Liviu Moldovan, Tiberiu Bataga

**Affiliations:** 1 Orthopedics—Traumatology Department, Faculty of Medicine, “George Emil Palade” University of Medicine, Pharmacy, Science, and Technology of Targu Mures, 540142 Targu Mures, Romania; 2 Quality Engineering Research Center, Faculty of Engineering and Information Technology, “George Emil Palade” University of Medicine, Pharmacy, Science, and Technology of Targu Mures, 540142 Targu Mures, Romania; liviu.moldovan@umfst.ro

**Keywords:** labor practices, sustainable development, reference framework, assessment, orthopedic, healthcare facility

## Abstract

*Background and Objectives*: The concept of sustainability in healthcare is poorly researched. There is a perceived need for new theoretical and empirical studies, as well as for new instruments to assess the implementation of new labor practices in the field. Such practices address unmet social needs and consolidate the sustainable development systems which promote health equity. The objective of the research is to design an innovative reference framework for sustainable development and health equity of healthcare facilities, and to provide a practical validation of this framework. *Materials and Methods*: The research methods consist of designing the elements of the new frame of reference, designing an indicator matrix, elaborating indicator content, and assessing the reference framework. For the assessment stage, we used sustainable medical practices reported in the scientific literature as well as a pilot reference framework that was implemented in healthcare practice. *Results*: The new reference framework suggested by the present study is composed of 57 indicators organized in five areas: environmental responsibility, economic performance, social responsibility, institutional capacity, and provision of sustainable healthcare services. These indicators were adapted and integrated into the seven basic topics of the social responsibility standard. The study presents the content of the indicators in the field of labor practices, as well as their evaluation grids. The innovative format of the evaluation grids aims to describe achievement degrees, both qualitatively and quantitatively. The theoretical model was validated in practice through its implementation at the Emergency Hospital in Targu Mures. *Conclusions*: The conclusions of the study reflect the usefulness of the new reference framework, which is compatible with the requirements in the healthcare field, but differs from other existing frameworks, considering its objective regarding the promotion of sustainable development. This objective facilitates the continuous quantification of the sustainability level, the promotion of sustainable development strategies, and sustainability-oriented approaches on the part of interested parties.

## 1. Introduction

Healthcare services have an intrinsic moral purpose, due to their inclusion of a series of labor practices that support people’s social needs. Labor practices comprise the entire set of policies and procedures related to activities performed within, by, or on behalf of the healthcare facility, including subcontracted works. The concept of labor practices extends beyond the relationship between a healthcare facility and its employees; it also addresses all the responsibilities of a healthcare facility in a workplace that it owns or directly controls. Healthcare organizations must improve the performance of the health system by implementing sustainable health equity frameworks which address unmet social needs. Such health equity frameworks have the potential to integrate and strengthen patient-centered labor practices in the organizational culture.

When evaluating public healthcare programs, a central criterion used by funders, including foundations, government agencies, and international agencies is sustainability [1]. Sustainability is defined as the deployment of human activities without destroying the environment and exhausting natural resources, therefore without compromising the prospects of future generations [2]. This is the essence of sustainable development that ensures a better life for current and future citizens of the planet [3]. In view of the above, community reference frameworks contribute to the support of dynamic and sustainable health systems. Thus, sustainability maybe considered an element of healthcare quality, by extending the responsibility of healthcare services offered to current and future patients.

At the community level, health policies aim to promote good health by addressing social needs, protecting citizens against threats, and supporting sustainable development. This can only be achieved by revolutionizing health systems with the help of new technologies, such as biotechnologies, genomics, e-health [4], and by ensuring the sustainability of these systems [5].

Although healthcare and medicine researchers have emphasized the issue of sustainability and health, the concept of sustainability in healthcare is still poorly researched, requiring new theoretical and empirical studies. Existing models of global healthcare focus on partnerships and lack sustainable integration [6]. Coiera [7] shows that the health system currently consumes enormous resources, generates enormous waste, and does not meet sustainability criteria. New interventions from outside the system are not sustainable approaches, because of the inability of outside designers to meet all the ever-evolving needs of those inside the medical system. There is a need to control the fulfillment level and the quality of the sustainability criteria. The legal frameworks and sustainability standards should include monitoring, reporting, verification, and transparency of the related regulations. Additionally, new tools must be developed to facilitate the evaluation of implementation efforts [8]. In a value-driven healthcare industry, it is imperative to facilitate service accessibility, improve patient experience and health outcomes, and reduce service costs. To achieve these results, it is essential to create new labor practices and develop new sustainability strategies.

The motivation for this research resides in the considerations above. Its main scope is to provide healthcare facilities with new tools for evaluating the quality and sustainability of labor practices, to support the management of healthcare facilities to discover new labor practices that address unmet social needs, and to elaborate on sustainable development strategies. The research aims to design an innovative framework for the assessment of sustainability and the promotion of sustainable development of healthcare facilities. This framework is compatible with national legislation, with national and international standards applicable in the field, and is validated through current hospital practice.

## 2. Materials and Methods

The exploratory research methodology applied in this study consists of:

1. Designing the new reference framework according to the 4 quality cycle steps, by integrating sustainable development requirements, social responsibility standards, and ensuring compatibility with the applicable legislation and standards in the health sector;
2. Exploring scientific medical literature and extracting scientifically confirmed evidence regarding the sustainability of medical practices;
3. Designing the indicator matrix by connecting the quality cycle core activities with the social responsibility core subjects based on successful practices described in the scientific literature;
4. Designing indicator content and evaluation grids based on the input of successful medical practices confirmed in scientific studies;
5. Implementing a pilot frame of reference in a hospital for the practical validation of the theoretical studies.

### 2.1. Areas of the New Reference Framework

In the first stage of the research, we explored the scientific literature in the medical field to identify the areas composing the new reference framework for the sustainable development of healthcare facilities. We intended to pinpoint a significant causal relationship between ensuring good institutional management and sustainability. Thus, the inclusion of the institutional area in the reference framework facilitates better management of the three areas of social, environmental, and economic sustainability, as shown in a study by Zdravkovic and Radukic [9].

Isaksson [10] shows that there is a symbiosis between quality management and sustainability, and healthcare reference frameworks must address these areas. For these reasons, along with the 4 components identified in the specialized literature, we incorporated a fifth one, pertaining to quality assurance of work practices. This fifth area is responsible for managing the basic processes of the healthcare facility.

**Figure 1:**
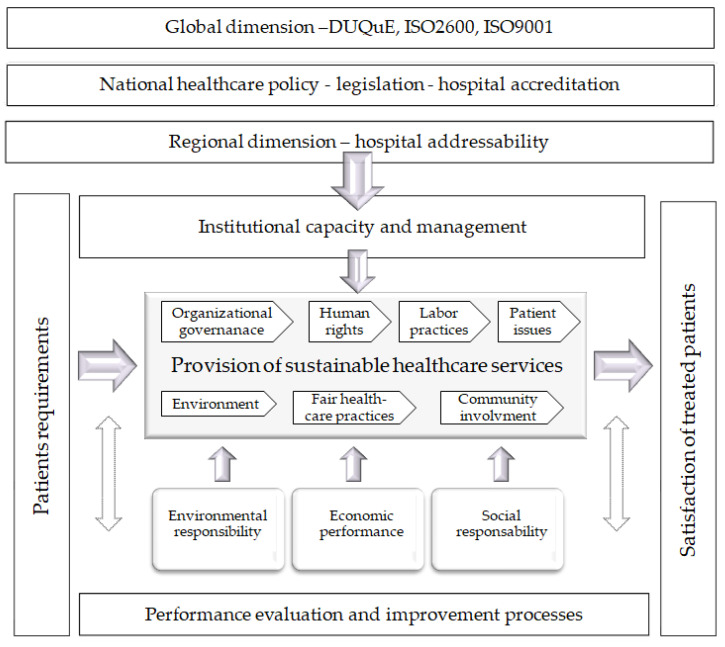
Conceptual model of the Health-Sustainability reference framework.

The conceptual model of the reference framework for Health-Sustainability (H-S) (Figure 1), comprises three sustainability areas: social, economic, and environmental. In addition to these, we added the area pertaining to organizational capacity and management. The area of sustainable healthcare services includes the 7 core subjects of the ISO 26000 standard on social responsibility, in an adapted form: organizational governance, human rights, labor practices, patient issues, environment, fair healthcare practices, and community involvement. 

The suggested model complies with standard global requirements in healthcare quality. It is compatible with the updated framework for evaluating and improving quality and safety in European hospitals DUQuE [11], ISO9001 quality management standards, national legislation, and the standards of A.N.M.C.S. (the authority that regulates quality assurance in the national health system in Romania) regarding hospital accreditation [12] and outpatient health services [13].

**Figure 2:**
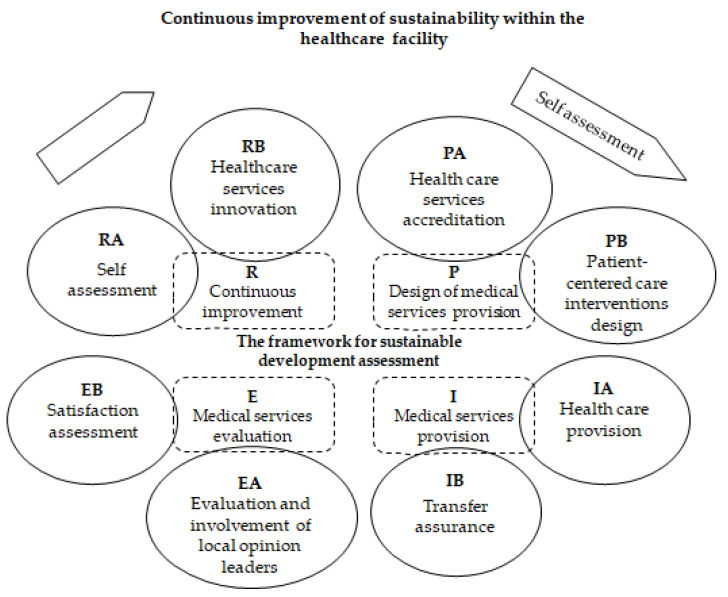
The sequence and interaction of the H-S core activities that make up the quality cycle.

In the next stage of the research, we designed two basic activities of the new reference framework for each stage of the quality cycle, using the elaborated conceptual model. These were adapted to the specifics of healthcare facilities regarding the provision of sustainable healthcare services [14]. Thus, in the first stage, we designed activities (PA)–Healthcare services accreditation and (PB)–Patient-centered care interventions design. In the implementation phase, we designed activities (IA)–Health care provision and (IB)–Transfer assurance. The evaluation phase consists of activities (EA)–Evaluation and involvement of local opinion leaders, and (EB)–Satisfaction assessment, while the review phase consists of activities (RA)–Self assessment, and (RB)–Healthcare services innovation. Figure 2 presents the structure of the quality cycle, the sequence, and interaction of the activities that compose it.

### 2.2. Evidence of Sustainable Labor Practices

In the next step of the theoretical study, we intended to elaborate on the content of the criteria and indicators composing the area of labor practices in the innovative reference framework. For this purpose, we sought confirmed evidence of best medical practices from representative medical institutions.

Consequently, we carried out a qualitative exploratory study of scientific publications from databases, especially PubMed, that presents new knowledge, evidence-based research, clinical studies, and organizational strategies. The research was carried out using keywords related to quality, sustainability, and labor practices, which was followed by a comparative analysis of the identified publications. Afterward, we extracted the contents which were relevant to the research objectives.

In the situations where we identified several solutions for the implementation of a certain sustainability-related requirement, we selected the more general one, which illustrates the evolution of sustainability within the system, from a simple continuous evaluation to sustainable development.

#### 2.2.1. Indicators for the Design of Medical Service Provision

Quality assurance processes in hospitals are classified into administrative-organizational processes and basic-professional processes [15]. As a result of hospital accreditation, the administrative-organizational processes become part of quality management [16], and their performance is in direct correlation with the 8 total quality management practices [17].

Labor practice quality in different medical specialties such as orthopedics-traumatology, together with survival rates in trauma [18], surgery and infection control [19], laboratory investigations, and acute myocardial infarction [20] are improved as a result of the hospital accreditation. In addition, mortality rates are low for patients treated in these units [21]. The meta-analysis carried out by Mansour et al. [22] based on the study of 78 articles shows that in low- and medium-income countries, accreditation has become a tool for improving the quality of medical services.

Professional development helps medical staff to improve their specialized knowledge and effectiveness. In order to achieve this aim, health units must adapt to a rapidly changing internal and external environment, with resource constraints and competitive medical assistance [23]. Managers who want to implement change, enhance quality, and promote teamwork in multidisciplinary healthcare settings can use learning from observation and practice [24]. Lifelong learning is an effective process [25], in which, through the integration of social networks, the medical staff stays up to date with practice guidelines, research, and skills [26]. In times of rapid and multiple changes, focusing on organizational development as a learning environment helps promote clinical nursing skills [27]. Appropriate professional and administrative support [28], expert involvement, clinical review processes, and practice opportunities can improve the quality of standard practice and professional development, leading to increased psychological well-being.

These activities collected from the scientific literature are inputs for the Promotion of change and professional development indicator. This indicator is related to the basic activity regarding the accreditation of healthcare services.

A number of randomized controlled trials and controlled clinical trials highlighted the effectiveness of patient-centered quality interventions, with remarkable results in the case of home health care [29]. Patient-centered care must focus on three broad areas of care: patient satisfaction, patient engagement, and care related to patient needs [30]. These approaches pursue three essential objectives: partnership, communication, and health promotion [31].

Patient-centered care strategies are implemented in hospitals even if the level of patient involvement in quality management functions is low [32]. The practice of patients’ access to their own electronic health records showed positive outcomes [33]. Asmat et al. [34] provided evidence supporting the effectiveness of patient-centered self-management care interventions in adults with diabetes.

Fossey et al. [35] demonstrated the effectiveness of training healthcare providers in patient-centered care. The use of informative materials and decision guides results in an extensive analysis of all of the patient’s health problems during the medical consultation. The quality of medical interventions is favored by empathy skills, patient-centered communication behavior, and data collection skills, but also by medical co-decision through patient involvement [36].

These activities collected from the scientific literature are inputs for the Quality assurance of patient-centered medical interventions indicator related to the basic activity regarding the patient-centered care intervention design.

#### 2.2.2. Indicators for Medical Service Provision

Medical education becomes effective only in a combination of traditional teaching methods (face-to-face courses), self-study (online courses), and ensuring access to databases with study material [37]. Continuous quality improvement is favored by continuous healthcare education, but the knowledge transfer between the staff participating in training and the rest of the staff is limited [38].

Studies show that improving medical care can be achieved through single or multiple interventions with the support of educational materials [39], reminders [40], through educational meetings [41], or through audits and feedback [42].

Studies [43,44] have shown that it is difficult to establish a direct relationship between organizational culture and the performance of the health system. Nevertheless, it is estimated that the clinical results of patients can be improved due to an increase in the level of organizational culture [45]. The strategies to improve the organizational culture can be standardized and made more efficient if they are supported by continuous healthcare education [46]. Additionally, a more comprehensive lifelong assessment of the physician’s competence will contribute to ensuring patients’ health [47]. These activities collected from the scientific literature are inputs for the Continuous healthcare education indicator, related to the basic activity regarding healthcare provision.

Similarly, another impact factor for hospital quality provision is hospital libraries. A hospital library functioning in a total quality management environment contributes to qualitypatient care. The result of the study by Fischer et al. [48] confirms the substantial clinical role of libraries and their cognitive significance. In addition to clinical libraries, clinical practice guidelines are sources of information for physicians and decision-makers. They have the potential to provide information for making high-quality patient decisions [49].

The effectiveness of disseminating and using clinical practice guidelines is well researched by numerous studies, which reported advancements in healthcare and suggested that these guidelines promote conformity with recommended standardized practices. Clinical practice guidelines have also been shown to be more useful when presented in portable, easily accessible formats and when used together with electronic reminders [40]. Very few studies have explored the costs of such dissemination strategies [41]. On the whole, dissemination of educational materials and short educational meetings seem to be the most feasible given the limited resources available [50]. Empirical evidence shows that using guidelines improves patient results, but adherence to the information they contain is fluctuant. For consistent results, active dissemination and innovative implementation strategies are required [51].

These activities collected from the scientific literature are inputs for the Practice guidelines employment and dissemination indicator, related to the basic activity regarding healthcare provision.

The effectiveness of interventions regarding the improvement of transfers reveals that the transfer solutions used, in addition to the potential to generate patient satisfaction, have the effect of avoiding adverse events [52]. Reducing adverse events can be achieved through effective communication and adequate planning [53]. The degree of information retention can be improved with the help of “clinical consensus statements” and “white papers” which have the role of supplementing verbal communication through the documented medium [54].

Education to improve transfers is reflected in the attitudes, knowledge, and skills of the medical staff involved in transfers. There is no concrete evidence regarding the improvement in patient outcomes [55].

Educational interventions influence knowledge, patients’ post-discharge emotional state, and medication adherence. A patient-tailored discharge plan in combination with post-discharge services provided at the patient’s home (e.g., in the case of the elderly diagnosed with heart failure) reduces the number of re-hospitalizations and may improve health results [56]. Patients who benefit from discharge planning have lower costs of laboratory services [57]. A lower re-admission rate is also recorded in the case of patients benefiting from the home hospitalization program [58]. When patient care trajectories are predictable, service improvement can be achieved through integrated care [59].

These activities collected from the scientific literature are inputs for the Interventions for transfers improvement indicator, related to the basic activity regarding transfer assurance.

#### 2.2.3. Indicators for Medical Service Evaluation

The results of the study by Roy et al. [60] show that “medical staff with professional skills” is the most important success factor in the quality management of hospital services. This means that the performance of the medical services provided by the hospital will increase with the recruitment of more qualified medical personnel.

Among the factors likely to influence the behavior of doctors and health professionals are: authoritative teaching, continuous traditional medical education, and influencers who can positively impact their colleagues [61]. Holliday et al. [62] describe the way of identifying influencers according to personal skills and manner of relating with others. Some evidence suggests that the amount of work and time allocated by influencers directly impacts the performance of clinical processes, through the quality and safety of the results obtained [63].

O’Brienet’s [64] meta-study looked at a wide variety of patient issues and concludes that professional practices benefit from mixed effects as a result of the involvement of local opinion leaders. Their intervention contributed to an increasing number of vaginal births after the patients had previously undergone a cesarean section, as well as a decrease in urinary catheter malpractice [64]. Community opinion leaders, combined with other educational programs, can successfully promote evidence-based medicine [65,66,67] and improve cancer pain management [68].

Rangachari [69,70] studied the organizational structure of knowledge sharing related to quality. He indicated that professional performance is improved by using a knowledge dissemination network that has a well-defined hierarchical structure, through which leaders make connections between certain groups of professionals, but also with the environment outside the hospital. Adversely, networks in which the connections between professionals are direct have lesser results. The interplay between individual opinion leaders, the collective process of negotiating change, and the re-orientation of professional norms is poorly understood, requiring a number of methodological concerns to be considered in further research in this area [71]. Rotar et al. [72] show that doctors taking over managerial roles and involving them in making strategic management decisions can lead to better-implemented quality management systems and an improvement in professional practices.

These activities collected from the scientific literature are inputs for the Professional practices improvement indicator, related to the basic activity regarding local opinion leaders’ evaluation and involvement.

A well-established and well-implemented quality management system leads to increased job satisfaction. Anyhow, the successful implementation of a quality management system requires commitment and support from the top management [73].

Quality management systems are more mature in hospitals that have a higher degree of social capital, and this favors successful cooperation and coordination within professional groups [74]. Medical staff satisfaction improves proportionally with the improvement of social capital, and this has direct effects on the quality of patient care [75].

Medical staff must assess the risk of burnout and can do this through individual and organizational resilience strategies [76]. Studying the opinions of the medical staff helps the hospital administration to develop plans to improve the organization of the staff’s work and to develop the network [77]. The factors that affect the satisfaction of the medical staff are the work itself, the work environment and atmosphere, the hospital management, the practice environment, and the rewards of the job [78]. McCay et al. [79] showed that relational leadership traits contribute to higher satisfaction of nursing staff, while task-oriented styles can reduce their satisfaction. Minimal information was found on the link between nursing staff leadership methods and patient satisfaction.

These activities collected from the scientific literature are inputs for the Medical staff satisfaction indicator, related to the basic activity regarding satisfaction assessment.

#### 2.2.4. Continuous Improvement Indicators

A series of experimental studies demonstrated some effects of audit and feedback on the professional improvement of health personnel [42]. It was demonstrated that feedback received from a colleague or superior is efficient when it is provided multiple times, both verbally and in written form [80], and if it indicates actions and improvement measures [81].

Two studies showed significant improvements in the desired outcome. In both cases, the interventions were positively received by the recipients of the feedback [82]. The first study was related to reducing inappropriate diagnoses of catheter-associated urinary tract infections in inpatient wards. In this case, the feedback intervention included elements that, according to feedback intervention theory, would best activate learning processes: framing feedback in terms of group performance and the provision of correct information about the solution. The second study implied the design of a web-based, report-style feedback intervention aiming to help primary care physicians improve their care of patients with hypertension. A study on clinical performance feedback formulates a few conclusions [83]: organizations and health professionals are limited in their capacity to interact with feedback, they have strong personal beliefs regarding the use of medical practices that restrict their availability of feedback, and the most effective feedback is the one which shapes clinical behavior.

These activities collected from the scientific literature are inputs for the Audit and feedback indicator, related to the basic activity regarding self-assessment.

The concept of “Lean healthcare” has been systematically introduced since 2006. The introduction of Lean principles into the care process can provide added value to the hospital’s quality management system, and hospital accreditation. External benchmarking has the potential to continuously improve the quality culture within the hospital [84].

A study that represents one of the first clinical applications of Six Sigma in surgery noted an improvement in sphincter preservation rates in rectal cancer patients by using a new Six Sigma surgical technique [85].

The effectiveness of Lean and Six Sigma is reported in a limited number of studies by reducing patient waiting times, improving infection control processes and antibiotic administration [86], reducing medication errors, and improving emergency medical care in operating rooms [87]. The use of Six Sigma leads to lower costs and significant savings [88]. Lean and Six Sigma methodologies contribute to optimizing the efficiency of the outpatient clinic and hospitalization times that can be reduced by up to three days for operated patients [89].

These activities collected from the scientific literature are inputs for the Medical organization supported by Six Sigma and Lean indicator, related to the basic activity regarding healthcare services innovation.

### 2.3. Description of Indicators and Evaluation Grids

In the next step of the research, we designed the 57 indicators of the new reference framework, by providing detailed descriptions in accordance with the successful practices confirmed in the scientific literature.

In order to facilitate the evaluation of the indicators, we formulated a basic set of questions for each indicator. The answers to the questions can be framed on a scale with 6 steps for evaluating the achievement degree of the indicator, quantified numerically by 0–5, and qualitatively by Not relevant–Low–Satisfactory–Good–Very good–Excellent.

The innovative evaluation methodology we propose in this research also evaluates the indicator importance, on a scale from 0 to 5, where 0—Not relevant; 1—Unimportant (subject of low importance for the organization); 2—Reduced importance (the organization’s activity is compromised by non-compliance with this requirement); 3—Important (the organization’s activity is affected by non-compliance with this requirement); 4—Very important (health care coverage is jeopardized by non-compliance with this requirement); 5—High importance (the organization’s existence is compromised by non-compliance with this requirement).

In this way, each indicator is evaluated numerically by a couple of values that describe the importance of the indicator and the achievement degree of the indicator, each of the two variables having values in the range of 0–5.

For the purposes of the present study, for reasons of space, we present in Appendix A (Table A1, Table A2, Table A3, Table A4, Table A5, Table A6, Table A7, Table A8, Table A9, Table A10, Table A11, Table A12, Table A13, Table A14, Table A15, Table A16, Table A17 and Table A18) the contents and innovative ways of evaluating the indicators that describe the responsibility related to labor practices: Table A1. The indicator PA3—Promotion of change and professional development; Table A2. Scale for indicator PA3—Promotion of change and professional development; Table A3. The indicator PB3—Quality assurance of patient-centered medical interventions; Table A4. Scale for indicator PB3—Quality assurance of patient-centered medical interventions; Table A5. The indicator IA31—Continuous healthcare education; Table A6. Scale for indicator IA31—Continuous healthcare education; Table A7. The indicator IA32—Practice guidelines employment and dissemination; Table A8. Scale for indicator IA32—Practice guidelines employment and dissemination; Table A9. The indicator IB3—Interventions for transfers’ improvement; Table A10. Scale for indicator IB3—Interventions for transfer improvement; Table A11. The indicator EA3—Professional practices improvement; Table A12. Scale for indicator EA3—Professional practices improvement; Table A13. The indicator EB3—Medical staff satisfaction; Table A14. Scale for indicatorEB3—Medical staff satisfaction; Table A15. The indicator RA3—Audit and feedback; Table A16. Scale for indicator RA3—Audit and feedback; Table A17. The indicator RB3—Medical organization supported by Six Sigma and Lean; Table A18. Scale for indicator RB3—Medical organization supported by Six Sigma and Lean.

For example, the indicator PA3—Promotion of change and professional development (Table A1) is described as: Identification of actions to promote change and improvement of the organizational structures of healthcare facilities that facilitate the improvement of the care process provided by medical assistance services. Identification of actions to promote professional development. Several questions are used for its evaluation: Are the needs to change the organizational structures identified? Are opportunities to improve healthcare services identified? Are the professional development requirements and opportunities of the medical staff identified? Is staff consulted to identify actions to promote change and professional development?

The Scale of the indicator PA3—Promotion of change and professional development (Table A2) contains the following scores/achievements: 1—Low: The promotion of change and professional development is a desire of the healthcare facility; 2—Satisfactory: Managerial analyses identify the needs to change the organizational structures. These changes are implemented according to the proposed action plans; 3—Good: Regular analysis of healthcare services, identification of improvement opportunities—implemented according to the proposed action plans; 4—Very good: Identification of the requirements and opportunities for professional development of the medical staff, design of professional development plans—implemented; 5—Excellent: The medical staff is regularly consulted to identify actions to promote change and professional development, and the results of the consultations are transposed into the action plans.

The main objective of the experimental part of the research was the practical validation of the H-S reference framework theoretical model carried out by implementing it at the Targu Mures County Emergency Hospital (ECHM) [90].The hospital has a high level of complexity due to the medical specialties, of which 57.33% are medical and 42.57% are surgical. It is included in the Mures Regional Emergency Functional Unit, with addressability for patients from the Center region.

Another goal of the implementation was to test and improve the content of the indicators and the evaluation questions. The duration of the evaluation was one week and was carried out by a group of four auditors. The selection criterion of the auditors was their involvement in the hospital’s quality assurance activities and the exercise of different medical tasks: head of the clinic, specialist doctor, medical assistant, and quality assurance manager.

The evaluation of the hospital focused on the three components of sustainability integrated into the core topics of social responsibility. We employed the innovative evaluation methodology designed in this research. According to this, for each indicator, the values regarding significance and degree of fulfillment were ranked on the designed scales from 0 to 5.

**Figure 3:**
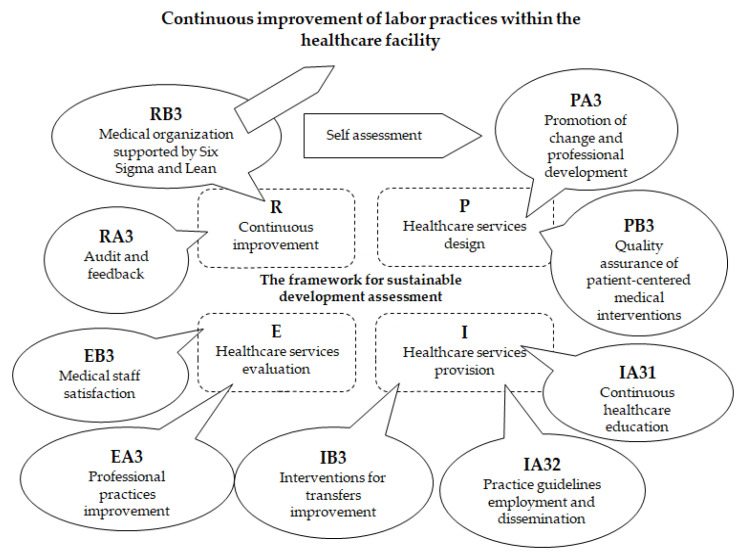
Thelabor practices continuous improvement cycle.

We evaluated labor practices within the healthcare organization, with the support of indicators described in Table A1, Table A2, Table A3, Table A4, Table A5, Table A6, Table A7, Table A8, Table A9, Table A10, Table A11, Table A12, Table A13, Table A14, Table A15, Table A16, Table A17 and Table A18 corresponding to the 4 phases of the quality cycle (Figure 3). In the planning phase, we used indicators PA3—Promotion of change and professional development and PB3—Quality assurance of patient-centered medical interventions. Next, in the implementation phase, we employed indicators IA31—Continuous healthcare education and IA32—Practice guidelines employment and dissemination and IB3—Interventions for transfer improvement. The evaluation continues in the next phase with indicators EA3—Professional practices improvement and EB3—Medical staff satisfaction. Lastly, in the review phase, we used indicators RA3—Audit and feedback and RB3—Medical organization supported by Six Sigma and Lean.

## 3. Results

The indicator matrix of the new reference framework for the sustainable development of health facilities was built on the basis of successful practices collected from the scientific literature. These practices indicated a connection between theeightbasic activities of the quality cycle (presented in the rows of Table 1) and the sevencore subjects of social responsibility (shown in the columns of Table 1). Whenever connections were identified, the need to develop an indicator was established. 

The names of the indicators were chosen so as to reflect the possible connection between quality cycle core activities and the social responsibility core subjects. If the successful practices derived from the literature indicated multiple activities, two indicators were designed. For example, indicators IA31—Continuous healthcare education and IA32—Practice guidelines employment and dissemination were designed to illustrate the connection between the activities of IA—Health care provision, andthe basic subject 3—Labor practices.

In the situations when the specialized literature did not identify successful activities, we have not provided an indicator. This is, for example, the case of the connection between the activity of EB—Satisfaction assessment and the core subject 4—Environment.

The result is the indicator matrix of the new reference framework for the sustainable development of health facilities, containing 57 indicators [14] (Table 1).

The present study summarizes below the findings and results recorded for the evaluation of the indicators composing responsibility related to labor practices.

PA3—Promotion of change and professional development—is supported by the development of medical scientific research, which is an integral part of hospital operations as a result of the development of medical-pharmaceutical teaching processes at different levels: high school, post-high school, university, and postgraduate. The teaching staff are integrated into the hospital or collaborate with the hospital.

The organizational structures of the hospital are presented in the organizational chart and are analyzed by the management. Consequently, hospital management identifies modification needs on a yearly basis. The strategies for managerial development and for the implementation of human resources are justified by the 4443 occupied positions and the 392 vacant positions. Medical education activities are carried out within the ECHM and are provided by the teaching staff of the “George Emil Palade” University of Medicine, Pharmacy, Science and Technology from Targu Mures (UMPST), which deploys integrated clinical activity. Within the hospital, a total of 1064 resident doctors (26.27% of the staff) complete their specialty training.

In 2021, the Ethics Commission for the Clinical Study of Medicine within the SCJUM approved a total of 258 applications for medical studies, in compliance with the rules in force, which support professional development opportunities of the medical staff.

PB3—Quality assurance of patient-centered medical interventions—the approved labor practices in each department of the institution have specific results. The follow-up of the protocol implementation is the responsibility of the quality manager, who is nominated within the department, the medical director, and the department head. The medical staff is regularly trained regarding approved medical protocols aimed at patient-centered care. They also receive instruction regarding the availability of newly designed and improved services, with clearly specified results.

IA31—Continuous healthcare education—an objective of human resources management is the professional development of all categories of personnel in order to increase the degree of professional training and maintain safe and effective professional performance. In order to achieve this objective, a total of 107 employees from different professional categories benefited from professional training internships.

In addition to practical medical activity, residents also attend postgraduate courses. Similarly, doctors attend a wide range of postgraduate training courses for various specialties, which are included in the UMPST curricula. Additionally, there are regular courses for medical staff, training activities, training services and clinical training for students and PhD students, as well as interdisciplinary collaborative events.

IA32—Practice guidelines employment and dissemination—based on the National Guidelines (e.g., Neonatology), these are updated according to European requirements. Clinical protocols are developed and used in current medical activities. Good practice guidelines are available (e.g., in intensive care) describing the structure of the clinic, job descriptions, protocols, specific managerial issues, and ethical issues.

The service for the prevention of intrahospital infections offers periodical training to medical staff. The training focuses on knowledge and respect of the legislation for the supervision and prevention of intrahospital infections, the legislation on cleaning, disinfection, and sterilization; information on the use of biocide products/disinfectants; medical waste management.

IB3—Interventions for transfers improvement—transfer effectiveness is improved with the support of electronic records and automatic updates facilitating effective access to and exchange of information. Continuity of care is ensured by involving patients and their families in the medical care process, and by the transmission of medical information and the treatment plan in an accessible form.

EA3—Professional practices improvement—the medical teams in the hospital wards include opinion leaders and experts in their specialist fields. They are recognized by specialist medical associations. Their involvement in specialist teams contributes to promoting appropriate professional behavior that leads to the improvement of medical services. An example in this respect is the application of orthopedic biomechanics methods [91].

EB3—Medical staff satisfaction—the employee satisfaction evaluation questionnaire, developed by the National Hospital Accreditation Commission, allows the collection of improvement opportunities suggested by employees. The questionnaire additionally tests the motivation for the medical profession, aspects related to institutional communication, professional development, continuous training, etc. The questions in the questionnaire were grouped and evaluated according to Maslow’s pyramid, in order to identify the major needs of the staff. The evaluations are periodical and quantify the evolution of staff satisfaction.

RA3—Audit and feedback—in the hospital, there is a structure and a body of auditors that periodically audit the quality management system according to the ISO9001:2015 reference. The clinical audit evaluates clinical practice in relation to existing and accepted medical standards. The clinical audit is also used as a self-assessment method to know the current situation and compare it to other medical practices. This is an opportunity to revise the care given to patients according to explicit criteria and to implement changes where necessary, with the potential to improve quality.

RB3—Medical organization supported by Six Sigma and Lean—The Lean vision of medical processes within the hospital is to transform a patient into a healthy person. Lean behavior aims to add value and reduce waste. Patient treatment is a trigger for a “pull” type process. The Lean vision is that the hospital should have standardized processes and working methods. Performance, which varies according to unpredictable factors, can be improved using Lean methods. The Lean approach starts with strategic measures and applies different specific improvement tools. Given the lack of a generalized Lean system, the practice relies on small and continuous changes that support Lean behavior. Such tools include Six Sigma, which quantifies medical service quality and the capability of healthcare processes.

The values of the indicators related to responsibility for labor practices are detailed in the self-evaluation instrument (Table 2).

**Figure 4:**
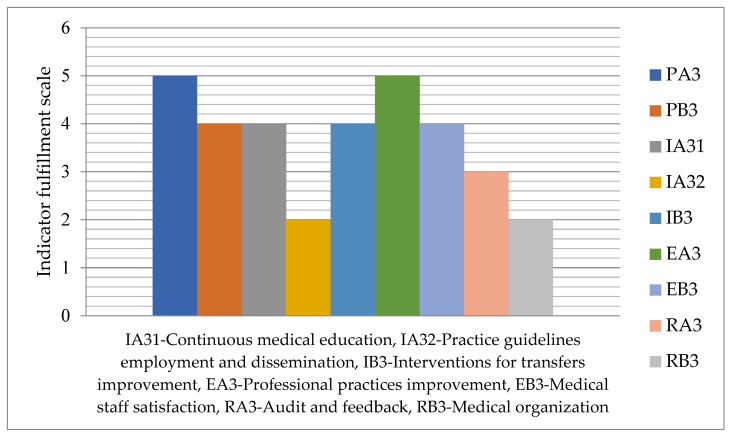
Fulfillment degree for labor practices responsibility indicators.

The degree of indicator fulfillment related to responsibility for labor practices, on a scale from 1 to 5, is graphically represented in Figure 4.

Two indicators, IA32—Practice guidelines employment and dissemination, and RB3—Medical organization supported by Six Sigma and Lean, have the degree of fulfillment 2, the lowest of this group, while the highest degree of fulfillment 5 is recorded for the other two indicators, PA3—Promotion of change and professional development, andEA3—Professional practices improvement.

**Figure 5:**
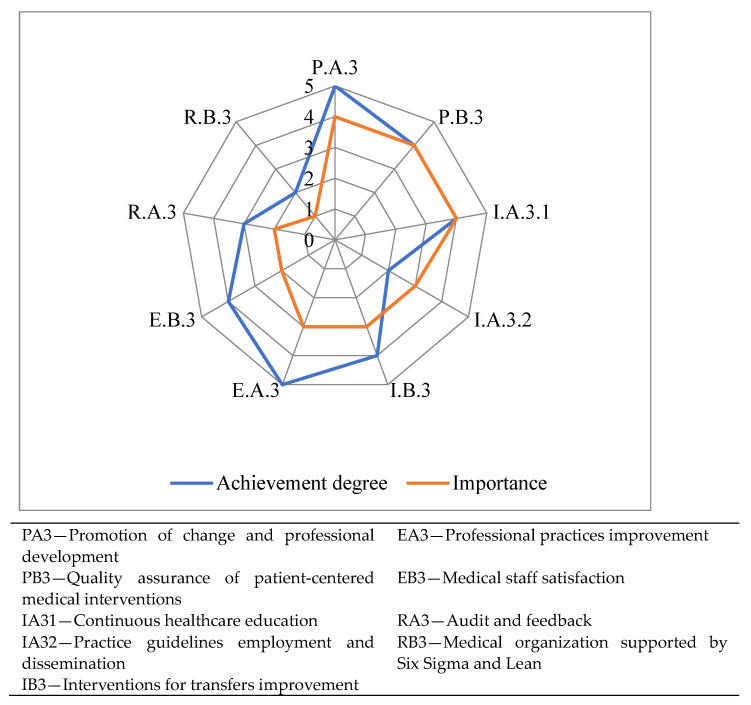
Labor practices sustainability evaluation graph.

The results for the performance indicators of responsibility for labor practices, in terms of importance and achievement degree, are represented in the labor practices sustainability evaluation graph (Figure 5).

The global sustainability indicator for labor practices (GS_LP_) is the sum of the ninesustainability indicators (Table 2):

(1) $${\mathrm{GS}}_{\mathrm{LP}}=\overset{9}{\underset{i=1}{\sum }}S_{i}=\overset{9}{\underset{i=1}{\sum }}I_{i}\cdot A_{i}=101$$

The maximal value of the Global Sustainability for labor practices (GSmax_LP_) is calculated for the maximum achievement score for every indicator:

(2) $${\mathrm{GSmax}}_{\mathrm{LP}}=5\cdot \overset{9}{\underset{i=1}{\sum }}I_{i}=5\cdot 26=130$$

The overall sustainability level of labor practices (LGS_LP_) is computed as a ratio between the current values and the maximal value of the indicator, expressed as a percentage:

(3) $${\mathrm{LGS}}_{\mathrm{LP}}=\frac{{\mathrm{GS}}_{\mathrm{LP}}}{{\mathrm{GSmax}}_{\mathrm{LP}}}\cdot 100=\frac{101}{130}\cdot 100=77.69\%$$

The value of the indicator characterizes the state of the hospital according to the requirements of the H-S reference framework for labor practices. 

**Figure 6:**
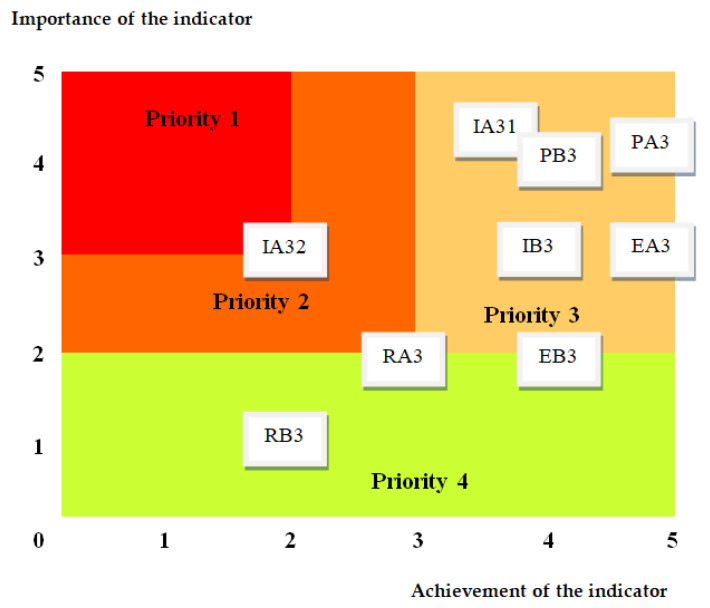
Labor practices sustainability assessment diagram.

Next, in order to improve the quality and sustainability assurance system, the results for responsibility related to labor practices are represented in an Eisenhower matrix. This includes a sustainability assessment diagram (Figure 6), which provides an overview of the sustainability level of labor practices.

With this support, depending on the fields where the indicators are located anddelimited by the scale symbolized by priority 1—high to priority 4—low, decisions are made to prioritize improvement measures.

From the analysis of the diagram, it follows that indicator IA32—Practice guidelines employment and dissemination must be treated with priority in order to improve the quality and sustainability of the labor practices responsibility.

## 4. Discussion

The pilot implementation and practical validation of the Health-Sustainability (H-S) reference framework was carried out at ECHM, which is an emergency hospital with a complex structure and a very high level of competence. The conceptual model of the new reference framework developed within this research was validated in practice at a microsystem level. This can be used as an example of good practice for other interested parties in the health managerial sector. In addition, the methodological approach can be used globally in any type of hospital or health center, and the results of the evaluations depend on the context in which each healthcare facility operates.

The participants demonstrated interest due to their individual concerns for sustainable development and due to the added value brought to the quality process by sustainable development issues. The experience was an opportunity to improve the culture of sustainability within the hospital, promoting sustainable and responsible behavior as well as equal opportunities through access to medical care.

The team of evaluators concluded that due to the multitude and complexity of the evaluated indicators, it was necessary to work in teams made up of several experts with professional experience and good knowledge of the basic processes of the hospital. The evaluation of the indicators could potentially lead to intense discussions regarding their interpretation or fulfillment degree. The success of the evaluation process depends on the way the chief auditor coordinates the evaluation team, but also on the efficiency of communication activities during the audit. Effective communication can stimulate the motivation of the audited personnel, who can provide relevant data about the department and/or the evaluated process. In this way, through the accuracy of the collected data, the evaluation process can be more efficient.

The evaluation experts highlighted the innovative character of the new reference framework, which allows the analysis of some aspects that the hospital does not evaluate regularly. The experts declared their satisfaction regarding participation in the pilot evaluation, which allowed them a much more complex analysis of the processes within the hospital.

It was found that the performance indicators of the H-S reference framework, designed on the basis of successful activities in medical practice, are adequate for evaluating the sustainability of the hospital. Through the pilot testing, some corrections were made in terms of the indicator content and the evaluation questions, so that they are as easy as possible to apply in subsequent evaluations. The experts recommended the design of a terminological glossary. It can facilitate a better mutual understanding for all participants in future evaluations.

The comparison of the reference framework developed in this research with other reference frameworks used in healthcare suggests that the H-S indicators are compatible with European DUQuE indicators [12]. The difference between the two reference frameworks is that H-S has, as its main objective, the promotion of sustainable development, which also distinguishes it in relation to other reference frameworks used in the health system. The comparison between H-S and other reference frameworks, whose objective is to ensure sustainable development, reveals the agreement between the results of the pilot evaluation with the help of H-S and other results of professional training organizations [92].

In our study, we proved that some medical practices improve once the hospital is accredited, due to the correlation with quality management activities. However, this does not lead to an improvement in patient satisfaction, as Sack et al. [16] also found. In addition, in accordance with the results of the study conducted by Groene et al. [32], we found that medical practices require better integration of patient-centered care across three broad domains: patient satisfaction, patient engagement, and care related to patient needs. Clinical decisions should be improved with the support of decision guidelines. These processes can be supported by the use of advanced electronic files using electronic systems, as other studies show [93,94]. These measures generate better satisfaction for the medical staff and the patients.

In our study, we found that continuing medical education can be better customized for participants with the support of clinical guidelines that contain hospital-specific aspects.

Improving fair transfer interventions by using the latest technical means and innovative training can reduce the number of adverse events at ECHM. This is supported by the scientific literature [52,54]. In addition, the effect of medical practices during recovery can be improved by collecting and assessing patient satisfaction and feedback, as reported by dePaula [95].

The evaluation of the sustainability of labor practices at ECHM suggests the necessity to improve labor practices by adopting the interventions suggested by local opinion leaders. A good capitalization of the audit results, together with the large-scale use of Six Sigma and Lean methods, can have the effect of improving medical organization.

This study has a series of limitations. In its current form, the new reference framework does not enclose all the institutional requirements of healthcare facilities regarding sustainability. It offers managers an opportunity to analyze organizational approaches related to quality assurance and sustainability implementation. One more limitation was generated by the practical validation of the reference framework that was carried out in an emergency hospital, which constitutes an extremely complex medical structure. It is necessary that, in the future, the indicators be tested and completed based on the results obtained at other healthcare facilities, covering a wide range of medical specialties, organizational dimensions, and forms of ownership.

Future research directions should be aimed at expanding the content of the reference framework to cover all organizational requirements, the development of a software tool that will help operationalize the system, and an evaluation methodology.

## 5. Conclusions

In this research, we developed a new reference framework for the sustainable development of health facilities (H-S). The indicators of the framework were designed based on new knowledge, evidence-based research, clinical trials, and government policies reported in scientific studies. The framework is compatible with the DUQuE reference framework developed for hospitals in the European community and with national hospital accreditation standards. The framework brings an additional contribution by promoting sustainability implementation and improvement in sanitary organizations.

H-S is a useful tool for the integration of sustainable development in the high-performance strategies that managers of healthcare facilities must develop in the current context of global challenges.

Based on the indicator system and the innovative methodology regarding evaluation, H-S establishes performance levels in the three dedicated areas of sustainability: economic, environmental, and social. Two additional areas are added: institutional capacity and medical assistance. This tool supports the design of a strategy for the sustainable development of the health facility, which is monitored through performance indicators.

The implementation of the new reference framework in healthcare facilities ensures respect for human rights, promotes the improvement of patient-centered labor practices, and guides medical staff and patients toward sustainable development.

## Figures and Tables

**Table 1:** The H-S framework indicator matrix.

Social Responsibility Quality Cycle		1−Organizational Governance	2−Human Rights	3−Labor Practices	4−Environment	5−Fair Healthcare Practices	6−Patient Issues	7−Community Involvement and Development
(P) Healthcare services design	PA−Healthcare services accreditation	PA1−Decision structures and processes	PA21−Health care services accessibility PA22−Medical care services for disadvantaged groups	PA3−Promotion of change and professional development	PA4−Plan for environmental impact	PA5−Attitudes of the profession towards accreditation	PA6−Performance information	PA7−Community involvement activities
	PB−Patient-centered care interventions design	PB1−Quality assurance processes design	PB2−Interventions with positive effects on patient satisfaction	PB3−Quality assurance of patient- centered medical interventions	PB4−Environmental criteria for the selection of materials used in interventions	PB5−Effective interventions implementation	PB6−Patient self-care design and self-management	PB7−Content of the interventions adapted to the community
(I) Healthcare services provision	IA−Health care provision	IA1−Computerized support systems for clinical decisions	IA2−Specific medical approaches	IA31−Continuous healthcare education IA32−Practice guidelines employment and dissemination	IA41−Usability of recycled materials IA42−Recycling of waste	IA5−Promotion of the patient safety culture	IA6−Critical features for improving the surveillance of patients with chronic conditions	IA71−Networking and partnership IA72−Involvement of volunteers and training networks
	IB−Transfer assurance	IB1−Transfer evaluation mechanisms	IB2−Fair transfer interventions	IB3−Interventions for transfers improvement	IB4−Environmentally friendly transfer interventions	IB5−Features that affect transfer effectiveness	IB6−Interventions to reduce problems in outpatients	IB7−Involvement and participation of professional associations
(E) Healthcare services evaluation	EA−Evaluation and involvement of local opinion leaders	EA1−Existence and recognition of local opinion leaders	EA2−Evaluation of current medical practices	EA3−Professional practices improvement	EA4−Environmental consumption improvement	EA5−Effective work practices	EA6−Patient-specific issues management	EA7−Local opinion leaders involved in the community
	EB−Satisfaction assessment	EB1−Monitoring mechanisms assignment	EB2−Patient satisfaction degree	EB3−Medical staff satisfaction	Not relevant	Not relevant	EB6−Patient satisfaction degree regarding therapeutic benefits	EB7−Satisfaction regarding partnerships
(R) Continuous improvement	RA-Self assessment	RA1−Self-assessment tools	RA2−Freedom of expression assurance	RA3−Audit and feedback	RA4−Waste generation and energy consumption surveillance tools	RA5−Feedback to medical staff	RA6−Complaints management	RA7−Communitarian initiatives
	RB−Healthcare services innovation	RB1−Changes to healthcare services	Not relevant	RB3−Medical organization supported by Six sigma and Lean	RB4−Measures applied to the environment	RB5−Safety checklists	RB6−Incident report	RB7−Educational visits

**Table 2:** Self-evaluation instrument for labor practices accountability.

No.	Indicator identifier	Importance Ii	Achievement Ai	Sustainability Indicator Si = Ii × Ai
1	PA3—Promotion of change and professional development	4	5	20
2	PB3—Quality assurance of patient-centered medical interventions	4	4	16
3	IA31—Continuous healthcare education	4	4	16
4	IA32—Practice guidelines employment and dissemination	3	2	6
5	IB3—Interventions for transfer improvement	3	4	12
6	EA3—Professional practices improvement	3	5	15
7	EB3—Medical staff satisfaction	2	4	8
8	RA3—Audit and feedback	2	3	6
9	RB3—Medical organization supported by Six sima and Lean	1	2	2

## Data Availability

The data used in this study can be requested from the corresponding author.

## Appendix A

**Table A1:** The indicator PA3—Promotion of change and professional development.

Indicator	PA3—Promotion of Change and Professional Development
Description	Identification of actions to promote change and improvement of the organizational structures of healthcare facilities that facilitate the improvement of the care process provided by medical assistance services. Identification of actions to promote professional development.
Evaluation questions	Are the needs to change the organizational structures identified? Are opportunities to improve healthcare services identified? Are the professional development requirements and opportunities of the medical staff identified? Is staff consulted to identify actions to promote change and professional development?

**Table A2:** Scale for indicator PA3—Promotion of change and professional development.

Score [A]	Achievement	Content
0	Not relevant	–
1	Low	The promotion of change and professional development is a desire of the healthcare facility.
2	Satisfactory	Managerial analyses identify the needs to change the organizational structures. These changes are implemented according to the proposed action plans.
3	Good	Regular analysis of healthcare services, identification of improvement opportunities—implemented according to the proposed action plans.
4	Very good	Identification of the requirements and opportunities for professional development of the medical staff, design of professional development plans—implemented.
5	Excellent	The medical staff is regularly consulted to identify actions to promote change and professional development, and the results of the consultations are transposed into the action plans.

**Table A3:** The indicator PB3–Quality assurance of patient-centered medical interventions.

Indicator	PB3—Quality Assurance of Patient-Centered Medical Interventions
Description	Designing interventions supported by medical professionals: - training the clinical consultant in patient-centered care; - providing decision guidance to improve clinical decision making.
Evaluation questions	How are patient-centered medical interventions designed? Is training of medical staff on patient-centered care taking place? Are decision guidelines provided to improve clinical decision making?

**Table A4:** Scale for indicator PB3—Quality assurance of patient-centered medical interventions.

Score [A]	Achievement	Content
0	Not relevant	–
1	Low	Some inputs can be used to design patient-centered medical interventions.
2	Satisfactory	There is an organizational framework that facilitates the design of patient-centered medical interventions.
3	Good	Patient-centered medical interventions are designed, and the designed services have output elements/specifications.
4	Very good	Regular staff training is conducted on patient-centered care and newly designed/improved services. Training includes the presentation of deliverables/specifications from the design stage.
5	Excellent	With the support of improved healthcare services, decision guidelines are developed and made available to medical staff to improve clinical decision-making.

**Table A5:** The indicator IA31—Continuous healthcare education.

Indicator	IA3—Continuous Healthcare Education
Description	Continuing medical education (CME) of health care professionals is intended to help professionals stay abreast of advances in patient care, adopt new, more beneficial care, and discontinue the use of costly diagnostic and therapeutic interventions that have limited benefit. CME tools and procedures include: vocational programs, collective education, professional information, clinical practice guidelines, reminders, opinion leaders’ interventions, audit and feedback. Continuing medical education improvement is supported by: - procedures for changing clinical practice and doctor behavior supported by trainers, reminders of optimal care, best practice information programs, guidelines of clinical practice and opinion leaders; - e-supported continuing medical education leads to changing practice patterns of sanitary personnel who can improve their competencies. Anyhow, if the program contains only plain text, it has limited effectiveness. The key characteristics that ensure success include the following: - the information transmitted by personalities; - targeting the interests and motivations of the group; - teamwork; - adapting the interventions to the needs of the audience; - collaborative learning; - the participation of top managers; - awareness of healthcare facility needs; - evidence of suboptimal use of health care; - convincing peers of the benefits of behavior change. There may be potential barriers to the application of clinical advances due to the rapid changes which occur, which can be stressful for doctors but also for potential patients. It is recognized that there are no unique professional education routes for every situation, and educators must select the appropriate methods according to the professional, social and economic particularities of the target group.
Evaluation questions	Is there a continuing medical education strategy/programme? What continuing medical education tools and techniques are used? The following elements are evaluated: vocational programs, collective education, professional information, clinical practice guidelines, reminders, opinion leaders’ interventions, audit and feedback. Are interactive programs on optimal versus effective care provided to practitioners and educators? Are there best practice outreach programs? Is electronic continuing medical education used? Is the information transmitted based on plain text only? What are the key characteristics that ensure the success of the applied continuing medical education program? The following elements are evaluated: information transmitted by personalities; targeting the interests and motivations of the group; teamwork; adapting the interventions to the needs of the audience; collaborative learning; the participation of top managers; awareness of the health unit’s needs; evidence of suboptimal use of health care; convincing peers of the benefits of behavior change. Do educators use approaches that focus on social, political, and economic particularities of the team and organization?

**Table A6:** Scale for indicator IA31—Continuous healthcare education.

Score [A]	Achievement	Content
0	Not relevant	–
1	Low	There is a strategy and plan for the implementation of continuing healthcare education programs.
2	Satisfactory	Continuing medical education programs are currently conducted with the support of continuing medical education tools and techniques, and the information conveyed is based on plain text only. These include vocational programs, collective education, professional information, clinical practice guidelines, reminders, opinion leaders’ interventions, audit and feedback.
3	Good	Information programs on best practices are conducted, and continuing medical education is supported electronically. Interactive programs on optimal versus effective care are offered to practitioners and educators.
4	Very good	The key characteristics that ensure the success of the continuous healthcare education program are identified, such as: the information transmitted by personalities; targeting the interests and motivations of the group; teamwork; adapting the interventions to the needs of the audience; collaborative learning; the participation of top managers; awareness of the needs of the healthcare facility; evidence of suboptimal use of health care; convincing peers of the benefits of practice change.
5	Excellent	In continuing medical education, educators use approaches that focus on social, political and economic particularities of the team and organization.

**Table A7:** The indicator IA32—Practice guidelines employment and dissemination.

Indicator	IA32—Practice Guidelines Employment and Dissemination
Description	By promoting evidence-based interventions, clinical guidelines improve patient care. Clinical guideline dissemination and implementation interventions consist of: - reminders (electronically or on paper); - dissemination of educational materials; - educational meetings; - patient-directed interventions. The effectiveness of clinical practice guidelines increases if they are presented in portable formats that are easy to access and use and are implemented using patient-specific reminders. Clinical practice guidelines are more effective if they are tailored to local needs.
Evaluation questions	Are evidence-based interventions promoted through clinical guidelines? What are the actions to disseminate and implement the clinical guidelines? The following elements are evaluated: reminders (in electronic format or on paper); dissemination of educational materials; educational meetings; patient-directed interventions. Are clinical guidelines presented in portable, easy-to-access formats? Are patient-specific reminders used? Are clinical practice guidelines adapted to local needs?

**Table A8:** Scale for indicator IA32—Practice guidelines employment and dissemination.

Score [A]	Achievement	Content
0	Not relevant	–
1	Low	Organization promotes evidence-based interventions with the support of clinical guidelines.
2	Satisfactory	Actions to disseminate and implement clinical guidelines aim at: reminders in electronic format or on paper; dissemination of educational materials; educational meetings; patient-directed interventions.
3	Good	Clinical guidelines are presented in portable, easy-to-access formats.
4	Very good	Patient-specific reminders are used.
5	Excellent	The clinical practice guidelines are adapted to local needs by covering the specific aspects identified.

**Table A9:** The indicator IB3—Interventions for transfers’ improvement.

Indicator	IB3—Interventions for Transfers’ Improvement
Description	Critical transition points in patient care during hospitalization and after discharge are called handoffs. They appear: - upon admission and discharge of the patient; - in intra-hospital transfer from one care provider to another. Incorrectly performed transfers can lead to adverse events and adverse influences on patients, therefore interventions aimed at optimizing hospital transfers must be applied. Interventions for improvement of intra-hospital transfers are: - the existence of a liaison nurse in intensive care units; - the use of transfer protocols, using the formula 1 stop-pit analogy and aviation expertise; - voicemail-based semi-structured sign-off for emergency department patients; - the presence of an accompanying pharmacist during the transfer of the patient from the oncology and hematology unit to critical care; - technological solutions, for example electronic templates that download information from medical databases; - adding written information to verbal information; - trainings related to information routes, error recognition, teamwork and communication using for example role play, observation, assessment and feedback.
Evaluation questions	Are transfers properly identified as critical transition points in patient care during and after hospitalization? Intra-hospital transfers, admissions and discharges of patients are evaluated. Is there a nurse liaison in intensive care units? Are transfer protocols used? They can be assessed using the Formula (1) pit stop analogy and aviation expertise. Is voicemail-based semi-structured sign-off used for patients picked up by the emergency department? During the transfer of the patient from the oncology and hematology unit to critical care is the presence of an accompanying pharmacist ensured? Are technological solutions used? For example, the existence of electronic templates that download information from electronic medical records is evaluated. Is verbal information supplemented by written information? Are educational interventions used for information management, error recognition, teamwork and communication? (using role play, observation, evaluation and feedback).

**Table A10:** Scale for indicator IB3—Interventions for transfer improvement.

Score [A]	Achievement	Content
0	Not relevant	–
1	Low	Intra-hospital transfers, admissions and discharges of patients are appropriately identified and treated as critical transition points in patient care during and after hospitalization.
2	Satisfactory	In intensive care units there is a liaison nurse. Transfer protocols exist and are enforced. Transfer protocols are evaluated by analogy with Formula 1 pit stops and aviation expertise.
3	Good	After discharge, continuity of care is planned to be achieved until the case is resolved. In the case of patients taken over by the emergency department, semi-structured disconnection based on voicemail is used. A pharmacist accompanies the transfer of the patient from the oncology and hematology unit to critical care.
4	Very good	Verbal information related to the transfer is supplemented with written information and technological solutions supported by IT systems are used to obtain information from electronic medical records.
5	Excellent	Transfers are improved through communication, teamwork, error recognition. Transfers are supported by information management training, using observation, role play, etc.

**Table A11:** The indicator EA3—Professional practices improvement.

Indicator	EA3—Professional Practices Improvement
Description	The practice of health professionals and their results are positively influenced by opinion leaders. Due to their status within the community and the credibility gained, they constitute a support for quality improvement strategies through: - transmitting the rules; - modeling convenient behavior; - dissemination of new technologies in the professional community.
Evaluation questions	Do local opinion leaders positively influence the practice of health professionals and their results? Are norms transmitted through the interventions of local opinion leaders? Do local opinion leaders contribute to modeling appropriate behavior? Is the use of new technologies disseminated among colleagues?

**Table A12:** Scale for indicator EA3—Professional practices improvement.

Score [A]	Achievement	Content
0	Not relevant	–
1	Low	The organization includes local opinion leaders that are recognized for their professionalism.
2	Satisfactory	Professional practice and health outcomes are positively influenced by local opinion leaders.
3	Good	Through the interventions of local opinion leaders, norms are transmitted and healthcare services are improved.
4	Very good	Local opinion leaders help shape appropriate behavior that results in improved healthcare services.
5	Excellent	The employment of innovative healthcare interventions is disseminated following the intervention of local opinion leaders, among professional colleagues.

**Table A13:** The indicator EB3—Medical staff satisfaction.

Indicator	EB3—Medical Staff Satisfaction
Description	The degree of satisfaction of the medical staff regarding the working conditions and the delivery of medical services.
Evaluation questions	Is the satisfaction of medical staff measured in terms of working conditions and medical service delivery? Compared to the previous assessment, how did the degree of satisfaction of the medical staff evolve?

**Table A14:** Scale for indicatorEB3—Medical staff satisfaction.

Score [A]	Achievement	Content
0	Not relevant	–
1	Low	There are updated questionnaires for evaluating medical staff satisfaction.
2	Satisfactory	Based on a procedure, questionnaires are periodically distributed to evaluate medical staff satisfaction.
3	Good	Periodically, the degree of medical staff satisfaction is measured in terms of working conditions and medical service delivery.
4	Very good	Compared to previous evaluation, there is an improved degree of satisfaction.
5	Excellent	Improvement measures are established to increase the satisfaction of the medical staff.

**Table A15:** The indicator RA3—Audit and feedback.

Indicator	RA3—Audit and Feedback
Description	Audit and feedback are essential quality improvement tools that can be used individually or in combination with other methods. Professionals are expected to improve activities when poor care outcomes and process deficiencies are highlighted. Audit and feedback processes are characterized by: - feedback format; - source of feedback; - feedback frequency; - improvement instructions; - type of change required; - the performance level to which they relate; - profession of the audited/recipient of feedback; - context; - targeted behavior. The effectiveness is greater or the improvement is more pronouncedin the following conditions: - the low level of performance to which it relates; - the sources of feedback collection are colleagues and superiors; - it is applied repetitively; - it contains quantifiable objectives, and an action plan for their achievement. The audit and feedback result is affected if: - results are communicated only verbally; - feedback is provided in graphic form without written text or action plan.
Evaluation questions	Is there a body of designated auditors? What is the frequency of audits? Is it performed repetitively? What performance level does the audit report to? Are the audit results documented? Do the conclusions contain the necessary direction of change? Do the conclusions contain explicit objectives and an action plan? In what format and how is feedback delivered? Does it consider the profession of the audited/feedback recipient? Is the context in which the audit is carried out considered? Is the behavior of the audit participants analyzed?

**Table A16:** Scale for indicator RA3—Audit and feedback.

Score [A]	Achievement	Content
0	Not relevant	–
1	Low	In the healthcare facility there is a body of designated auditors, who have the necessary level of training to carry out audits.
2	Satisfactory	There is an annual audit plan that establishes the frequency of audits in each compartment. The audit plan establishes the performance level to which the audit reports.
3	Good	The audit results are documented, and the audit conclusions contain proposals for improving the activity in the audited department.
4	Very good	Based on the proposals made to improve the activity, an action plan is drawn up with explicit objectives, activities, responsibilities and allocated resources.
5	Excellent	The behavior of the participants in the audit and the context in which the audit is carried out are analyzed. The format and mode of transmission of audit feedback takes into account the profession of the audited/recipient.

**Table A17:** The indicator RB3—Medical organization supported by Six Sigma and Lean.

Indicator	RB3—Medical Organization Supported by Six Sigma and Lean
Description	Continuous improvement in the quality of healthcare can be achieved with the help of data provided by Six Sigma and Lean. Both tools use data and quantitative methods to improve quality and ensure progress towards an assumed goal. Combined use provides processes focused on measuring and eliminating errors (Six Sigma) and ensuring an efficient and value-added workflow (Lean). They facilitate improved care processes and clinical outcomes, as well as the economic efficiency of healthcare organizations.
Evaluation questions	Is the Lean method used to continuously improve the quality of healthcare? Is the Six Sigma method used to continuously improve the quality of healthcare? What are the errors measured and eliminated using the Six Sigma method? Can you describe the extent to which the efficiency and added value of the work flow has increased by applying the Lean method?

**Table A18:** Scale for indicator RB3—Medical organization supported by Six Sigma and Lean.

Score [A]	Achievement	Content
0	Not relevant	–
1	Low	Data are collected and quantitative methods are used to improve quality and ensure progress towards the stated goal.
2	Satisfactory	The healthcare facility uses Six Sigma and Lean methods in a cycle of continuous improvement of the medical care quality.
3	Good	The combined use of the two methods provides processes focused on measuring and eliminating errors (Six Sigma) and ensuring an efficient and value-added workflow (Lean).
4	Very good	The use of Six Sigma and Lean in the medical organization results in improved clinical outcomes, care processes and financial performance of the healthcare facility.
5	Excellent	Data are collected and quantitative methods are used to improve quality and ensure progress towards the stated goal.
